# Supplementary material for: Association of IRS1 (Gly972Arg) and IRS2 (Gly1057Asp) genes polymorphisms with OSA and NAFLD in Asian Indians
Source: PLoS One. 2021 Aug 27;16(8):e0245408. doi: 10.1371/journal.pone.0245408 (PMC8396739; doi:10.1371/journal.pone.0245408)
Supplement: S1 Table — (DOCX) [file pone.0245408.s001.docx]

S1 Table: Association of IRS-1 gene polymorphism with clinical, body composition, anthropometry and biochemical parameters

| **Variables** | **OSA with NAFLD**  **(n=130** | | | **OSA without NAFLD**  **(n=100)** | | | **Without OSA with NAFLD**  **(n=95)** | | | **Without OSA and without NAFLD (n=85)** | | |
| --- | --- | --- | --- | --- | --- | --- | --- | --- | --- | --- | --- | --- |
|  | **Gly/Gly** | **Gly/Arg** | **Arg/Arg** | **Gly/Gly** | **Gly/Arg** | **Arg/Arg** | **Gly/Gly** | **Gly/Arg** | **Arg/Arg** | **Gly/Gly** | **Gly/Arg** | **Arg/Arg** |
| Age (yrs.) | 45.8±9.4 | 45.7±9.5 | 45.8±9.7 | 44.9±12.9 | 44.5±10.5 | 45.4±11.5 | 42.6±11.3 | 38.9±11.2 | 34.8±15.7 | 41.2±9.2 | 41.9±10.2 | 42.4±10.0 |
| SBP (mmHg) | 132.8±11.9 | 133.4±15.6 | 134.9±16.7 | 129.9±8.1 | 131.2±10.6 | 134.1±10.5 | 128±8.5 | 130.3±9.1 | 132.4±16.8 | 126.1±11.9 | 129.2±9.3 | 130.2±8.7 |
| DBP (mmHg) | 85.6±24.1 | 85.9v23.7 | 86.0±7.6 | 83.8±7.0 | 84.6±6.4 | 86.7±6.7 | 83.8±6.5 | 84.3±7.2 | 85.4±9.02 | 82.4±3.9 | 83.2±7.4 | 84.9±7.0 |
| BMI (kg/m²) | 32.1±4.3 | 35.6±7.6 | 40.0±6.4^*^ | 31.6±6.3 | 32.6±5.1^*^ | 34.8±5.3^*^ | 31.3±6.6 | 32.5±9.1 | 33.6±9.7 | 30.3±4.2 | 31.2±31.2 | 32.4±32.5 |
| Fat mass (kg) | 36.0±16.9 | 45.5±15.6 | 57.6±24.4^*^ | 29.2±13.7 | 34.2±5.8 | 37.0±13.5 | 35.5±16.5 | 35.3±15.2 | 35.8±14.9 | 28.7±12.2 | 30.9±11.2 | 31.2±8.5 |
| FFM (kg) | 54.4±12.2 | 53.4±11.4 | 52.8±12.0 | 53.9±10.6 | 50.3±12 | 48.0±14.0 | 46.7±11.4 | 46.7±11.2 | 45.6±11.3 | 47.9±12.0 | 45.8±11.2 | 44.8±8.9 |
| Body fat (%) | 37.3±11.6 | 38.6±7.6 | 40.7±8.8^*^ | 34.1±11.3 | 39.8±13.0 | 43.4±15.0 | 33.1±13.1 | 37.8±11.2 | 42.6±12.8 | 38.2±12.8 | 36.7±4.6 | 34.9±13.5 |
| WC (cm) | 105.5±9.8 | 106.4±12.3 | 107.6±15.5 | 105.2±10.8 | 107.3±11.1 | 110.1±11.8 | 102.6±11.0 | 105.3±15.4 | 106.3±16.6 | 101.1±5.9 | 102.1±9.8 | 102.4±8.6 |
| HC (cm) | 105.5±11.5 | 107.3±15.4 | 109.8±17.9 | 106.8±12.2 | 107±12.1 | 109±12.3 | 103.8±10.5 | 105.4±9.2 | 107.3±18 | 95.5±28.3 | 102.8±26.8 | 105.8±10.3 |
| MTC (cm) | 56.9±13.0 | 57.4±12.2 | 58.1±16.0 | 47.6±9.7 | 51.3±10.4 | 55.3±10.4 | 52.8±11.9 | 51.2±11.6 | 52.7±11.8 | 46.5±8.7 | 47.8±15.6 | 48.3±29.7 |
| MAC (cm) | 27.4±5.2 | 28.5±3.2 | 30.6±6.8 | 29.5±6.9 | 29.8±6.3 | 30.7±7.3 | 28.3±4.9 | 28.7±5.4 | 28.4±5.6 | 26.8±6.6 | 27.8±20.3 | 28.3±20.1 |
| NC (cm) | 38.6±6.0 | 38.6±4.1 | 39.1±3.9 | 37.7±3.7 | 37.2±5.9 | 38.5±3.6 | 36±3.9 | 36.8±5.4 | 37±4.3 | 34.2±3.6 | 35.1±3.7 | 35±3.1 |
| Biceps (mm) | 16.8±7.1 | 16.5±6.3 | 16.7±6.9 | 14.5±6.4 | 15.9±7.9 | 18.8±7.2 | 17.3±21.3 | 17.2±4.5 | 17.5±5.6 | 14.7±5.2 | 15.6±6.5 | 16.5±6.5 |
| Triceps (mm) | 24.9±10.0 | 24.6±10.2 | 25.1±10.2 | 22.5±8.1 | 26.4±15.9 | 30.4±11.3 | 21.0±8.5 | 25.3±10.2 | 26.7±11.3 | 23.8±8.4 | 24.3±7.6 | 24.5±7.7 |
| Sub Scapular (mm) | 28.9±6.7 | 30.2±13.2 | 34.2±8.9 | 25.9±6.0 | 34±6.7 | 36±6.1 | 25.9±7.8 | 26.7±11.2 | 31.3±13.2 | 27.7±5.6 | 32.5±5.4 | 34.2±6.9 |
| Suprailiac (mm) | 31.9±8.7 | 31.4±9.7 | 31.3±9.4 | 28.2±5.9 | 29.8±6.5 | 30.5±9.1 | 28.7±7.4 | 31.2±6.7 | 32.3±11.0 | 30±6.5 | 34.7±11.2 | 36.7±12.5 |
| LT (mm) | 33.3±9.1 | 36.7±9.5 | 41.1±55.2 | 29.6±7.0 | 34.6±12.4 | 38±8.3 | 31.4±8.8 | 33.5±9.8 | 36.6±13.9 | 32.5±9.6 | 31.2±14.6 | 33.4±11.0 |
| Thigh (mm) | 25.2±12.2 | 25.6±9.3 | 26.0±9.3 | 24.8±7.9 | 25.1±6.7 | 25.8±6.5 | 24.4±8.3 | 25.6±8.3 | 26.2±7.8 | 29.3±13.3 | 30.9±6.3 | 32±5.7 |
| FBG (mg/dl) | 98.8±15.2 | 102±14.5 | 105.0±27.4^*^ | 93.1±19.8 | 102±13.4 | 107.2±42.0 | 97.8±23.1 | 98.2±11.2 | 99.2±12.0 | 76.5±42.6 | 79.0±11.2 | 94.6±15.0 |
| TG (mg/dl) | 170.5±90.5 | 180.6±54.2 | 213.0±81.2^*^ | 156.8±69.6 | 165.3±71.2 | 170±71.4 | 161.5±63.4 | 163.6±4.2 | 166.8±43.3 | 148.2±75.7 | 154.9±14.3 | 157±86.2 |
| TC (mg/dl) | 181.7±50.6 | 183±54.2 | 189.1±42.9^*^ | 177.5±36.1 | 181.2±24.5 | 182.0±23.3 | 167.8±27.5 | 170.2±34.5 | 176.0±37.6 | 156. 7±26.6 | 165.9±34.5 | 177±44.11 |
| HDL-C (mg/dl) | 42.6±7.9 | 41.9±9.3 | 41.6±9.2 | 42.9±7.2 | 44.2±8.3 | 46.8±21.4 | 45.2±9.8 | 44.5±6.5 | 42.3±5.5 | 44.6±6.5 | 57.9±7.6 | 71.7±78.0 |
| LDL-C (mg/dl) | 112.9±45.2 | 111.9±32.2 | 111.6±34.2 | 102.4±33.0 | 94.5±25.6 | 86.7±16.2 | 113.1±38.3 | 101±23.2 | 93.4±21.8 | 115.7±19.4 | 111.9±36.7 | 95±61.2 |
| ALT (IU/L) | 40.9±30.7 | 45.6±21.3 | 51.2±29.9^*^ | 27.2±12.7 | 34.5±14.5 | 40.6±18.5 | 40.2±20.6 | 40.2±19.2 | 40.4±20.0 | 40±32.8 | 52.1±6.7 | 56.2±43.2 |
| AST (IU/L) | 45.3±30.1 | 47.6±32.1 | 54±36.7^*^ | 30.0±15.7 | 36.5±13.4 | 41.7±13.2 | 51.3±40.4 | 51.2±32.1 | 52.0±46.3 | 32.5±26.8 | 37.8±4.8 | 46.7±32.9 |
| Insulin (µU/ml) | 10.2±8.0 | 11.3±6.2 | 12.2±7.1^*^ | 11.3±5.2 | 10.5±3.5 | 10.5±2.8 | 9.3±4.2 | 9.3±4.1 | 9.2±4.2 | 8.4±3.6 | 10.1±9.8 | 11.6±15.3 |
| HOMA-IR | 1.8±0.5 | 1.9±0.6 | 2.1±0.6^*^ | 1.8±0.9 | 1.5±0.5 | 1.7±0.8 | 1.67±0.1 | 1.75±0.6 | 1.5±0.5 | 1.6±0.5 | 1.5±3.2 | 1.4±0.5 |

Results are shown as mean± SD. P value is <0.05 is statistically significant. ^*^ Represent p value <0.05. SBP, systolic blood pressure; DBP, diastolic blood pressure, WC, waist circumference; BMI, body mass index; FFM, fat free mass; HC hip circumference; MTC, mid thigh circumference; MAC, mid arm circumference; NC, neck circumference; LT, Lateral Thoracic; FBG, fasting blood glucose; TG, triglyceride; TC, total cholesterol; HDL, high density lipoprotein; VLDL, very low density lipoprotein; ALP, Alkaline phosphate; ALT, alanine transaminase; AST, aspartate transaminase; HOMA-IR, homoeostasis model assessment of insulin resistance.

Table 2: Association of IRS-2 gene polymorphism with clinical, body composition, anthropometry and biochemical parameters

| **Variables** | **OSA with NAFLD**  **(n=130** | | | **OSA without NAFLD**  **(n=100)** | | | **Without OSA with NAFLD**  **(n=95)** | | | **Without OSA and without NAFLD (n=85)** | | |
| --- | --- | --- | --- | --- | --- | --- | --- | --- | --- | --- | --- | --- |
|  | **Gly/Gly** | **Gly/Asp** | **Asp/Asp** | **Gly/Gly** | **Gly/Asp** | **Asp/Asp** | **Gly/Gly** | **Gly/Asp** | **P** | **Gly/Gly** | **Gly/Asp** | **Asp/Asp** |
| Age (yrs) | 46.5±10.5 | 44.9±10.2 | 45.7±10.2 | 43.6±11.6 | 44.6±13.5 | 45±14.5 | 42.6±14.6 | 39.5±14.5 | 41.2±4.5 | 41.5±9.5 | 43.5±12.3 | 41.2±10.2 |
| SBP (mmHg) | 131.9±10.9 | 133.2±15.6 | 133.4±9.3 | 130±9.2 | 132.5±9.5 | 133.4±10.2 | 129±9.5 | 131.5±15.6 | 130.4±15.6 | 129.5±10.9 | 131.5±9.6 | 132.4±10.3 |
| DBP (mmHg) | 87.6±22.5 | 85.6±16.5 | 86.7±15.3 | 84.6±6.2 | 86.9±6.7 | 86.3±7.7 | 84.6±6.5 | 85.4±9.2 | 86.7±24.5 | 83.4±3.0 | 84.9±8.0 | 85.6±10.2 |
| BMI (kg/m²) | 35.8± 6.4 | 36.7±6.7 | 39.9±11.5 | 31.9±6.2 | 34.1±5.9 | 34.1±6.7 | 30.7±6.5 | 35.6±9.1 | 34.5±12.3 | 30.1±3.6 | 35.1±10.5 | 32.6±11.4 |
| Fat mass (kg) | 39.6±10.6 | 41.4±7.8 | 42.1±419.5 | 30.9±10.8 | 33.6±13.2 | 32.1±8.9 | 34.7±16.2 | 37.5±16.0 | 35.7±13.4 | 29.4±13.2 | 30.3±8.3 | 30.2±12.2 |
| FFM (kg) | 53.8±11.5 | 53.5±12.3 | 54.2±13.5 | 52.6±10.5 | 50.9±9.8 | 51.2±6.8 | 47.9±9.5 | 45.8±10.5 | 46.8±9.8 | 45.6±6.9 | 46.5±7.9 | 46.7±7.8 |
| Body fat (%) | 35.6±10.6 | 39.8±13.2 | 41.6±12.5 | 35.6±14.6 | 42.6±12.6 | 41.2±13.2 | 35.6±14.5 | 38.5±12.5 | 38.9±12.0 | 39.8±15.6 | 40.5±10.5 | 40.5±10.2 |
| WC (cm) | 104.6±30.2 | 105.6±12.3 | 106.5±25.6 | 106.5±24.6 | 109.5±22.3 | 108.3±12.9 | 103.6±36.5 | 107.5±26.5 | 105.7±5.9 | 101.5±12.6 | 102.6±13.5 | 102.3±34.8 |
| HC (cm) | 106.5±25.6 | 107.8±21.2 | 110.5±32.6 | 106.5±13.5 | 109.5±11.3 | 109.8±21.2 | 104.6±15.6 | 106.5±10 | 105.7±10.1 | 100.2±13.6 | 104.6±12.3 | 104.4±13.2 |
| MTC (cm) | 56.9±13.0 | 58.1±16.0 | 58±15.1 | 49.6±9.7 | 54.3±10.4 | 52.3±16.7 | 53.6±11.9 | 54.6±11.8 | 52.3±11.9 | 47.5±8.7 | 48.3±29.7 | 47.8±12.3 |
| MAC (cm) | 27.6±6.3 | 28.5±7.2 | 28.9±6.5 | 30.5±6.4 | 31.5±6.6 | 31.9±21.2 | 29.6±4.9 | 28.9±5.6 | 28.9±6.1 | 27.1±6.6 | 28.6±4.9 | 27.9±3.2 |
| NC (cm) | 37.5±5.6 | 39.4±5.6 | 39.8±6.6 | 36.5±6.5 | 37.5±7.5 | 38.9±4.5 | 35.6±8.6 | 36.5±9.5 | 35.7±10.2 | 34.5±12.6 | 35.1±10.2 | 35.6±14.6 |
| Biceps (mm) | 15.6±5.6 | 16.4±5.6 | 16.7±5.4 | 14.5±3.5 | 16.5±4.6 | 15.6±4.7 | 13.5±5.6 | 15.6±6.3 | 15.4±6.1 | 14.7±5.6 | 15.6±6.3 | 15.4±7.8 |
| Triceps (mm) | 24.1±9.0 | 24.9±10.2 | 25.6±10.1 | 23.5±7.1 | 25.6±10.2 | 24.6±5.8 | 21.6±9.5 | 25.6±9.8 | 24.8±21.1 | 24.6±6.5 | 26.5±9.5 | 26.7±12.3 |
| Sub Scapular (mm) | 29.6±11.6 | 32.5±14.6 | 31.2±13.2 | 28.5±13.2 | 30.5±12.3 | 31.2±13.4 | 26.5±9.8 | 28.5±8.5 | 27.8±13.1 | 27.5±6.5 | 31.2±5.3 | 30.2±6.5 |
| Suprailiac (mm) | 31.5±9.5 | 33.6±10.6 | 32.4±10.2 | 29.5±5.6 | 31.2±6.1 | 30.2±6.5 | 28.6±7.1 | 31.5±4.6 | 30.1±6.9 | 27.9±5.9 | 30.5±6.1 | 30.1±6.5 |
| LT (mm) | 38.6±12.1 | 40.2±16.5 | 39.8±14.2 | 32.5±5.6 | 36.4±6.3 | 33.5±7.2 | 31.2±2.6 | 35.5±3.2 | 32.1±2.6 | 31.5±2.6 | 32.6±3.2 | 32±12 |
| Thigh (mm) | 25.9±11.2 | 26.5±10.3 | 26.8±10.2 | 24.3±10.3 | 25.1±10.5 | 25.6±24.2 | 24.6±9.6 | 26.5±10.5 | 24.7±9.2 | 24.6±10.3 | 25.6±9.5 | 25.6±10.2 |
| FBG (mg/dl) | 98.5±26.5 | 104.5±25.6 | 103.1±23.4 | 95.6±24.6 | 102.3±23.6 | 101.1±19.9 | 96.5±25.4 | 98.5±24.6 | 99.7±18.9 | 78.5±16.5 | 83.5±15.6 | 84.5±15.6 |
| TG (mg/dl) | 185±75.6 | 203±81.2 | 199.7±91.2 | 161.3±46.5 | 169.8±23.6 | 168.3±23.1 | 161.5±26.5 | 163.2±24.6 | 162.8±31.1 | 148.2±26.5 | 152.3±32.6 | 151.2±31.2 |
| TC (mg/dl) | 180.2±45.6 | 187.3±46.5 | 188.3±43.2 | 178.6±22.6 | 181.4±21.5 | 182.3±19.3 | 169.5±25.6 | 175.6±36.5 | 174.2±32.1 | 166.9±22.1 | 169.5±23.6 | 167.2±26.7 |
| HDL-C (mg/dl) | 41.56±12.5 | 40.23±12.6 | 39.1±13.4 | 42.5±13.5 | 45.4±12.5 | 45.6±14.3 | 45.5±13.5 | 44.6±12.5 | 43.2±14.5 | 43.5±12.6 | 44.5±13.5 | 44.6±12.5 |
| LDL-C (mg/dl) | 112.6±50.1 | 113±48.6 | 112.4±12.3 | 101.5±26.5 | 96.5±24.2 | 101.4±29.8 | 110.3±23.6 | 95.4±25.6 | 105.2±28.7 | 112±30.6 | 100.5±30 | 114.1±30 |
| ALT (IU/L) | 45.1±12.6 | 52.5±13.6 | 52.3±12.7 | 27.8±12.6 | 38.4±16.5 | 39.8±12.3 | 38.9±12.5 | 41.1±16.9 | 41±15.1 | 38.5±14.6 | 41.2±12.3 | 42.5±12.3 |
| AST (IU/L) | 41.5±10.2 | 49.2±11.5 | 46.8±13.4 | 32.5±9.5 | 40.5±10.3 | 38.9±10.2 | 49.5±9.5 | 51.6±14.6 | 50±5.2 | 42.5±19.6 | 45.8±16.4 | 43.8±13.8 |
| Insulin (µU/ml) | 9.56±3.2 | 11.9±3.5 | 10.5±4.5 | 9.5±2.6 | 10.4±3.5 | 9.9±1.9 | 8.2±2.5 | 9±2.4 | 9.3±2.8 | 8.2±3.4 | 10.5±2.3 | 9.8±3.5 |
| HOMA-IR | 1.98±1.0 | 1.9±0.9 | 1.97±0.7 | 1.87±0.8 | 1.81±0.9 | 1.83±0.98 | 1.65±0.6 | 1.56±0.8 | 1.6±0.5 | 1.50±0.7 | 1.45±0.8 | 1.4±0.5 |

Results are shown as mean± SD. P value is <0.05 is statistically significant. SBP, systolic blood pressure; DBP, diastolic blood pressure, WC, waist circumference; BMI, body mass index; FFM, fat free mass; HC hip circumference; MTC, mid thigh circumference; MAC, mid arm circumference; NC, neck circumference; LT, Lateral Thoracic; FBG, fasting blood glucose; TG, triglyceride; TC, total cholesterol; HDL, high density lipoprotein; VLDL, very low density lipoprotein; ALP, Alkaline phosphate; ALT, alanine transaminase; AST, aspartate transaminase; HOMA-IR, homoeostasis model assessment of insulin resistance.
